# Intermittent Fasting and Emotional Regulation: A Psychobiological Framework Integrating Metabolic, Neuroendocrine and Interoceptive Mechanisms

**DOI:** 10.3390/nu18101626

**Published:** 2026-05-20

**Authors:** Ettore D’Aleo, Mara Lastretti, Tiziano Scarparo, Emanuela A. Greco, Andrea Cicoli, Sabina Spagna, Gavino Faa, Lorenzo Campedelli

**Affiliations:** 1 Department of Economic, Psychological and Communication Sciences, Niccolò Cusano University, 00191 Rome, Italy; mara.lastretti@unicusano.it (M.L.); info@tizianoscarparo.it (T.S.); andrea.cicoli@unicusano.it (A.C.); sabina.spagna@unicusano.it (S.S.); lorenzo.campedelli@unicusano.it (L.C.); 2 Department of Medical Sciences and Public Health, University of Cagliari, 09124 Cagliari, Italy

**Keywords:** intermittent fasting, interoception, emotional regulation, psychobiology, metabolic switching, neuroendocrine adaptation, heart-rate variability, stress response, eating behavior, psychological vulnerability

## Abstract

**Background/Objectives**: Intermittent fasting (IF) has been widely investigated for its metabolic effects, including improvements in insulin sensitivity, lipid metabolism, and inflammatory markers. However, its psychological and experiential dimensions remain comparatively underexplored. The present narrative review examines IF within a psychobiological framework, integrating evidence from metabolic science, neuroendocrinology, and affective neuroscience to explore its potential impact on emotional regulation and interoceptive processes. **Methods**: A structured narrative literature search was conducted across PubMed, Scopus, and Google Scholar, focusing on studies published between 2010 and 2025. Eligible studies included human and relevant animal research addressing metabolic, hormonal, interoceptive, and psychological responses to IF. Evidence was synthesized thematically to identify convergent mechanisms linking metabolic adaptations to emotional and regulatory outcomes. **Results**: Available literature suggests that IF is associated with a metabolic shift toward lipid utilization, characterized by increased ketone body production, particularly β-hydroxybutyrate. These adaptations appear to be accompanied by modulation of neuroendocrine pathways and may influence central nervous system functioning through mechanisms potentially related to neuroinflammation, mitochondrial efficiency, and synaptic plasticity. Emerging evidence further suggests that IF may modulate BDNF signaling and gut–brain axis activity, although direct causal pathways in humans remain to be established. At the psychological level, IF is associated with heterogeneous emotional outcomes: structured fasting protocols have been linked to modest improvements in perceived stress and mood in metabolically healthy individuals, whereas irritability, anxiety, or behavioral rigidity may emerge in those with pre-existing psychological vulnerabilities. Individual differences in interoceptive sensitivity, emotion regulation strategies, and moderating biological factors—including sex, circadian timing, and habitual physical activity—appear to influence these responses. **Conclusions**: Overall, IF may be conceptualized as a context-dependent psychobiological stressor whose effects extend beyond metabolic regulation to include interoceptive and emotional processes. These effects appear bidirectional, potentially promoting psychological resilience in some individuals while increasing the risk of affective destabilization or maladaptive behaviors in others. Current evidence remains limited by a lack of integrative and longitudinal studies combining metabolic and psychological measures. Future research adopting multidisciplinary approaches is needed to clarify the mechanisms underlying individual variability and to better define the potential benefits and risks of IF in both clinical and non-clinical populations.

## 1. Introduction

In recent years, intermittent fasting (IF) has attracted increasing attention within both scientific and public health contexts. Initially investigated for its metabolic effects, IF has been associated with improvements in insulin sensitivity, lipid metabolism, and inflammatory markers, and has progressively been explored in relation to aging, cancer prevention, and cognitive function [1,2]. A key metabolic feature of IF is the shift from glucose-based energy utilization toward increased reliance on lipid substrates, including free fatty acids and ketone bodies. This metabolic transition reflects enhanced lipolysis and ketogenesis and is considered a central component of fasting-related physiological adaptation. Short fasting intervals that do not induce a measurable shift toward lipid utilization are therefore not the primary focus of the present review.

Despite the growing body of literature on metabolic outcomes, relatively less attention has been devoted to the psychological and experiential dimensions of IF. Most studies have primarily focused on biochemical and physiological markers, with limited investigation of how fasting-related metabolic changes may interact with subjective experience, emotional regulation, and internal bodily awareness. However, emerging evidence suggests that fasting may influence not only metabolic processes but also the perception and regulation of internal states, which may in turn affect adherence, tolerability, and overall outcomes. Recent narrative syntheses have further highlighted that IF encompasses heterogeneous paradigms—including time-restricted eating, alternate-day fasting, periodic prolonged fasting, and religious or ritual fasting—and that the current literature remains disproportionately centered on metabolic endpoints, with comparatively limited integration of psychological and psychiatric outcomes [3].

From a psychobiological perspective, IF may be conceptualized as a controlled metabolic challenge that engages neuroendocrine pathways influencing both peripheral physiology and central nervous system functioning. These processes include modulation of the hypothalamic–pituitary–adrenal (HPA) axis, changes in autonomic regulation, and increased ketone body availability, all of which have been associated with effects on stress responsivity and neural function [4,5]. In this context, lipid-derived substrates are increasingly recognized not only as energy sources but also as signaling molecules involved in neuroinflammatory pathways, mitochondrial function, and synaptic plasticity. In particular, β-hydroxybutyrate has been proposed as a key mediator linking metabolic changes to central processes, acting as both an energetic substrate and a signaling molecule involved in epigenetic modulation, neurotrophic pathways, and inflammatory regulation [6].

Within this framework, interoception—the perception and integration of internal bodily signals—represents a potential interface linking metabolic changes to emotional and behavioral regulation. Interoceptive processes are mediated by distributed neural networks, including the insular cortex and anterior cingulate cortex, and contribute to the monitoring of physiological states such as hunger, arousal, and fatigue [7,8]. Rather than acting as a direct causal mechanism, interoception may modulate how metabolic and endocrine signals are interpreted at the subjective level.

Current evidence suggests that IF may alter interoceptive signaling, with potentially heterogeneous psychological effects. Some individuals report improved regulation of internal states, whereas others may experience increased irritability, anxiety, or cognitive rigidity, particularly in the presence of pre-existing vulnerabilities [9,10]. These findings indicate that the psychological impact of fasting is not uniform, but may depend on individual differences in interoceptive sensitivity, stress reactivity, and emotion regulation strategies. Importantly, recent clinical syntheses describe fasting as a bidirectional intervention, potentially associated with modest improvements in mood and perceived stress under structured conditions, but also with affective destabilization or restrictive behaviors in vulnerable individuals [11].

In light of these considerations, the present narrative review aims to examine IF within an integrated psychobiological framework, with particular attention to the interaction between lipid metabolism, neuroendocrine adaptation, and interoceptive processes. The objective is not to provide clinical recommendations, but to synthesize current evidence and identify potential mechanisms that may account for variability in emotional and psychological responses to fasting.

The review first considers IF as a form of adaptive metabolic stress, and subsequently examines its potential effects on interoception, emotional regulation, and psychological vulnerability. Finally, key limitations in the current literature are discussed, together with directions for future research integrating metabolic and psychological domains.

## 2. Materials and Methods

### 2.1. Study Design

This work was conducted as a narrative review aimed at integrating evidence across metabolic science, neuroendocrinology, and clinical psychology to explore the psychobiological effects of intermittent fasting (IF). Given the conceptual and interdisciplinary scope of the topic, a narrative approach was considered appropriate to identify and synthesize emerging mechanisms linking metabolic adaptations to interoceptive and emotional processes.

Although the review was not designed as a systematic review or meta-analysis, efforts were made to enhance methodological transparency and reproducibility in the identification, selection, and synthesis of the literature, in line with recent recommendations for narrative evidence synthesis in this field [12].

### 2.2. Search Strategy

A structured literature search was performed between July and December 2025 using the electronic databases PubMed/MEDLINE, Scopus, and Google Scholar. Google Scholar was included as a supplementary source to capture interdisciplinary contributions not always indexed in biomedical databases. To mitigate the risk of selection bias inherent to this database, its use was restricted to the identification of peer-reviewed studies with clear methodological rigor or seminal theoretical contributions in psychology and psychophysiology. Studies retrieved exclusively through Google Scholar were included only when they met the same eligibility criteria applied to PubMed/MEDLINE and Scopus sources, and priority was consistently assigned to results from indexed databases.

Search terms were combined using Boolean operators and included the following keywords: “intermittent fasting”, “time-restricted feeding”, “metabolism”, “ketone bodies”, “interoception”, “interoceptive awareness”, “emotional regulation”, “stress response”, “HPA axis”, “anxiety”, and “eating disorders”.

The search was limited to articles published in English. Priority was given to studies published between 2010 and 2025, while earlier seminal works were included when directly relevant to interoceptive or neuroendocrine mechanisms. In addition, reference lists of relevant articles were manually screened to identify further eligible studies.

### 2.3. Eligibility Criteria

Studies were considered eligible if they met at least one of the following criteria:

(1) Investigated intermittent fasting or time-restricted feeding in human populations;
(2) Examined metabolic or hormonal responses associated with fasting, including lipid metabolism, ketogenesis, or endocrine adaptations;
(3) Explored interoceptive processes, autonomic regulation, or psychophysiological responses;
(4) Reported psychological or behavioral outcomes, including emotional regulation, mood, anxiety, or vulnerability traits.

Both experimental and observational studies, as well as relevant narrative and systematic reviews, were included. Animal studies were considered when they provided mechanistic insights directly applicable to human psychobiological processes.

### 2.4. Exclusion Criteria

Studies were excluded if they:

(1) Focused exclusively on weight loss, body composition, or athletic performance without addressing psychophysiological or regulatory mechanisms;
(2) Involved fasting paradigms not consistent with intermittent or time-restricted feeding models;
(3) Lacked sufficient methodological detail;
(4) Consisted of editorials, commentaries, or non-peer-reviewed material;
(5) Reported exclusively metabolic outcomes without relevance to psychological, interoceptive, or behavioral processes.

### 2.5. Study Selection and Data Extraction

Titles and abstracts were initially screened to assess relevance. Full-text articles were subsequently evaluated based on the eligibility criteria.

For each eligible study, key information was extracted, including study design, population characteristics, fasting protocol (type and duration), metabolic and hormonal outcomes, and psychological or interoceptive measures. Where available, validated psychometric instruments and indicators of stress physiology were also considered.

Given the narrative nature of the review, no formal quantitative quality scoring system was applied; however, greater interpretive weight was assigned to peer-reviewed studies with clearly defined fasting protocols and validated psychological or psychophysiological outcomes, in line with current methodological considerations in the field.

### 2.6. Data Synthesis

The selected literature was synthesized using a thematic approach. Studies were grouped according to recurring conceptual domains, including:

(1) Metabolic and neuroendocrine adaptations to IF;
(2) Interoceptive processing and bodily awareness;
(3) Emotional regulation and psychological variability;
(4) Clinical vulnerabilities and potential risks.

The aim of this synthesis was not to quantify effect sizes, but to identify convergent patterns and propose an integrative framework linking metabolic signals to subjective and emotional processes.

### 2.7. Methodological Considerations

Due to the narrative design, this review does not provide a quantitative assessment of evidence or a formal risk-of-bias analysis. As such, the findings should be interpreted as a conceptual integration of current knowledge rather than as definitive conclusions. To enhance methodological transparency, a qualitative classification of evidence was applied throughout the synthesis. Randomized controlled trials and pre-registered prospective studies were considered to provide the strongest basis for causal inference; observational and cross-sectional studies were treated as supporting evidence; and narrative reviews, theoretical papers, and case reports were included for contextual integration only. Throughout this review, a deliberate effort has been made to distinguish between relationships supported by direct empirical evidence in humans, those inferred from animal or mechanistic studies, and those that remain primarily theoretical or hypothetical. Readers are encouraged to attend to this distinction when evaluating the proposed psychobiological framework.

However, particular attention was paid to heterogeneity in fasting protocols, study populations, and outcome measures, as well as to the distinction between self-reported and clinician-assessed psychological outcomes, which may influence the interpretation of results. Future studies would benefit from standardized outcome measures, systematic adverse-event reporting, and longer follow-up periods to enhance comparability and clinical applicability [11].

The following provides a transparent account of the literature selection process. Records were identified through structured searches of PubMed/MEDLINE and Scopus, supplemented by Google Scholar for interdisciplinary contributions. Following title and abstract screening against the eligibility criteria defined in Section 2.3 and Section 2.4, full-text articles were retrieved and assessed; those not meeting the inclusion criteria were excluded. Greater interpretive weight was assigned to studies with clearly defined fasting protocols and validated psychological or psychophysiological outcomes, in line with the qualitative evidence hierarchy described in Section 2.7. The final set of included studies formed the basis of the thematic narrative synthesis presented in the Results. A formal quantitative flow diagram (PRISMA) was not produced, as this format is designed for systematic reviews; however, the selection rationale is documented transparently across Section 2.1, Section 2.2, Section 2.3, Section 2.4, Section 2.5, Section 2.6 and Section 2.7 to enable methodological appraisal by the reader.

### 2.8. Classification of Fasting Paradigms

The main fasting paradigms addressed in this review differ substantially in their temporal structure, metabolic profile, and psychological implications. Given the importance of distinguishing between these protocols when interpreting the available evidence, a brief structured classification is provided below: (1) Time-Restricted Eating (TRE): caloric intake confined to a daily window of 4–10 h, with the remaining hours spent in a fasted state; metabolic effects depend on window duration and circadian alignment. (2) Alternate-Day Fasting (ADF): alternation between unrestricted eating days and complete or near-complete fasting days (0–25% of energy needs); more pronounced metabolic and neuroendocrine effects compared to TRE. (3) Periodic Prolonged Fasting: fasting periods of 24–72 h or more, typically performed once or twice per month; associated with more robust ketogenic responses and greater HPA axis activation. (4) 5:2 Protocol: five unrestricted eating days combined with two non-consecutive days of severe caloric restriction (approximately 500–600 kcal/day). (5) Religious and Ritual Fasting (e.g., Ramadan): culturally embedded fasting patterns with specific temporal constraints; effects are influenced by social context, circadian disruption, and intention. Where studies do not distinguish between these paradigms, this limitation is noted in the text and evidence is interpreted with appropriate caution.

## 3. Results

### 3.1. Intermittent Fasting as a Metabolic and Neuroendocrine Stressor

The findings of the included studies are synthesized below according to key thematic domains. Available evidence suggests that intermittent fasting (IF) is associated with a metabolic shift from glucose-based to lipid-based energy utilization, characterized by increased lipolysis, elevated circulating free fatty acids, and enhanced ketone body production [1,13]. These changes appear to be accompanied by reduced insulin levels and modulation of cortisol secretion, reflecting coordinated endocrine adaptations to energy restriction. It should be noted that the strength and consistency of these findings vary across fasting paradigms: more pronounced metabolic shifts have been reported with alternate-day fasting and prolonged fasting protocols, whereas time-restricted eating may produce more modest effects depending on window duration and timing [14]. In particular, ADF protocols have been most consistently associated with robust ketogenic responses and HPA axis activation, while the 5:2 protocol produces an intermediate metabolic profile; TRE effects are strongly modulated by the timing of the eating window relative to the circadian phase. These paradigm-specific differences should be considered when interpreting the findings synthesized in the following sections [15].

Several studies have reported that short-term fasting is associated with transient activation of the hypothalamic–pituitary–adrenal (HPA) axis, resulting in moderate increases in circulating cortisol levels [4,5]. Importantly, this response appears to be time-limited and may support metabolic adaptation rather than reflecting chronic stress activation. The magnitude of this response has been shown to vary according to circadian timing, sex, and individual stress reactivity [16].

In parallel, ketone bodies, particularly β-hydroxybutyrate, have been identified as signaling molecules involved in the regulation of inflammation, oxidative stress, and gene expression [6]. Experimental and clinical studies suggest that these metabolic changes may influence central nervous system functioning through mechanisms related to mitochondrial efficiency and synaptic plasticity [17,18]. Importantly, these neurobiological effects remain primarily characterized in preclinical models, and direct causal evidence in humans is still limited.

Beyond energy metabolism, emerging evidence suggests that fasting-associated ketogenesis may influence central neurobiological mechanisms through additional pathways. β-hydroxybutyrate has been proposed to upregulate brain-derived neurotrophic factor (BDNF), a neurotrophin involved in synaptic plasticity, neurogenesis, and mood regulation [19]. BDNF signaling may therefore represent a potential mechanistic bridge between fasting-induced metabolic changes and improvements in cognitive and affective functioning reported in some studies; however, this pathway has been primarily characterized in preclinical models and its translation to human psychological outcomes requires further investigation. In addition, the vagus nerve plays a key role in transmitting interoceptive signals from peripheral organs, including the gastrointestinal tract, to central circuits involved in emotional regulation [20]. Fasting-related changes in gut microbial composition may interact with vagal afferent pathways, potentially modulating interoceptive processing and affective states through the gut–brain axis [21]. While these mechanisms are supported by growing preclinical and observational evidence, they should currently be considered as plausible hypothetical pathways rather than established causal mechanisms in humans.

In addition to endocrine responses, IF has been associated with markers of metabolic flexibility and cellular adaptation, including improved mitochondrial function and activation of autophagic pathways [12]. These findings support the view that IF may act as a mild and time-limited metabolic challenge associated with adaptive physiological responses.

Importantly, emerging human evidence suggests that these physiological adaptations may also translate into measurable changes in stress-related systems. Supervised fasting interventions have been associated with reductions in basal cortisol levels and increases in heart-rate variability, indicating improved autonomic regulation and stress resilience [22].

However, the extent to which these metabolic and endocrine changes translate into consistent psychological or behavioral effects remains unclear, highlighting the need for integrative approaches that consider both biological and psychological variables.

### 3.2. Endocrine–Psychological Interactions in Intermittent Fasting

Evidence indicates that intermittent fasting (IF) is associated with a range of endocrine adaptations that extend beyond metabolic regulation and may influence psychological and behavioral processes. Hormonal responses to fasting appear to contribute to the modulation of internal bodily states, which are relevant for emotional and interoceptive processing.

One of the primary systems involved is the hypothalamic–pituitary–adrenal (HPA) axis. Short-term fasting has been associated with moderate increases in cortisol secretion, particularly during early phases of metabolic switching [4,5]. While chronic elevations in cortisol are linked to adverse psychological outcomes, transient increases observed during fasting may support metabolic adaptation and energy mobilization [23]. However, individual variability in HPA axis responsivity may influence how these endocrine changes are experienced at the psychological level.

Changes in insulin and leptin levels also contribute to the systemic and central effects of fasting. Reduced insulin concentrations facilitate lipolysis and ketogenesis, while also affecting central pathways involved in reward processing and energy regulation [13]. Leptin, in addition to its role in appetite regulation, has been implicated in mood and stress-related processes. Lower leptin levels during fasting have been associated with increased irritability and affective instability in some populations [24].

Ghrelin represents another hormone of interest in the context of fasting-related psychobiological responses. Circulating ghrelin levels typically increase during fasting and have been shown to influence hunger perception, reward sensitivity, and stress-related behaviors [25]. Experimental evidence suggests that ghrelin may exert context-dependent effects, potentially supporting stress adaptation in some conditions while contributing to increased anxiety or emotional reactivity in others. Importantly, the magnitude of ghrelin elevation varies substantially across fasting paradigms: ADF and prolonged fasting protocols tend to produce greater and more sustained ghrelin elevations compared to TRE, which may partly explain differences in hunger-related emotional reactivity reported across protocols.

Ketone bodies, particularly β-hydroxybutyrate, have been identified as signaling molecules with potential effects on brain function. In addition to their role as alternative energy substrates, ketones have been associated with modulation of inflammatory pathways, oxidative stress, and synaptic plasticity [6]. Some studies suggest that these mechanisms may support cognitive function and mood regulation, although findings remain variable and dependent on individual and contextual factors [18].

From a psychophysiological perspective, endocrine changes during fasting are closely linked to interoceptive processing. Hormonal fluctuations influence bodily sensations such as hunger, fatigue, and arousal, which are integrated within neural networks involving the insular cortex and anterior cingulate cortex [8,9]. Variability in interoceptive sensitivity may therefore contribute to differences in emotional responses to fasting.

Importantly, clinical evidence suggests that these endocrine and metabolic adaptations may be associated with heterogeneous psychological outcomes. Structured fasting interventions have been linked to modest improvements in depressive symptoms and perceived stress in metabolically healthy individuals, whereas less controlled or prolonged fasting may increase vulnerability to irritability, anxiety, or affective instability in at-risk populations [26].

Overall, current evidence suggests that endocrine adaptations to IF are associated with heterogeneous psychological outcomes. While some individuals may experience improved regulation of internal states, others may report increased irritability or emotional instability. These differences appear to be influenced by individual factors, including stress reactivity, interoceptive processing, and psychological vulnerability.

Further research integrating endocrine, metabolic, and psychological measures is needed to clarify the mechanisms underlying these associations and to better characterize individual variability in response to fasting.

### 3.3. Interoception as a Bridge Between Metabolism and Mind

Interoception, defined as the perception and integration of internal bodily signals, has been increasingly recognized as a central construct in affective neuroscience and psychophysiology [7,9]. Interoceptive processes enable the detection of physiological changes such as hunger, thirst, fatigue, and autonomic arousal, thereby contributing to emotional awareness, self-regulation, and the integration of bodily states into subjective experience.

From a neuroanatomical perspective, interoception is supported by a distributed network including the posterior and anterior insular cortices, anterior cingulate cortex, brainstem nuclei, and somatosensory pathways [8]. Within this system, the insular cortex plays a key role in integrating visceral and metabolic inputs and generating representations of internal bodily states relevant for behavioral and emotional regulation [19].

In the context of intermittent fasting (IF), interoceptive signaling appears to be modulated, particularly during prolonged fasting periods. Variations in hunger, energy availability, autonomic activity, and circulating metabolic substrates may increase the salience of internal bodily signals. Some studies suggest that fasting may be associated with changes in interoceptive accuracy, defined as the ability to detect and interpret internal signals, although current evidence remains limited and context-dependent [20]. It should be noted that interoceptive changes appear particularly pronounced during ADF and prolonged fasting protocols, which involve more extended periods of caloric restriction and thus greater fluctuations in metabolic signals. TRE protocols may produce less marked interoceptive perturbations, though circadian alignment of the eating window may independently influence autonomic and interoceptive functioning.

A distinction between interoceptive awareness and somatic hypervigilance is particularly relevant in this context. Interoceptive awareness refers to a non-judgmental and adaptive engagement with internal bodily states and has been associated with more effective emotion regulation. In contrast, somatic hypervigilance involves heightened, anxiety-driven monitoring of bodily sensations and has been linked to anxiety disorders and related conditions [21,22]. This distinction may be especially relevant during fasting, when physiological signals are amplified.

Variability in interoceptive processing may contribute to heterogeneous psychological responses to IF. In some individuals, increased awareness of bodily signals may support improved regulation of internal states, whereas in others similar physiological changes may be experienced as distressing, particularly in the presence of elevated anxiety sensitivity or reduced interoceptive confidence.

Interoceptive processes are also closely linked to autonomic nervous system (ANS) regulation. Fasting-related metabolic changes may influence the balance between sympathetic and parasympathetic activity, with downstream effects on arousal and stress responsivity [23]. Alterations in vagal tone, in particular, have been associated with differences in emotion regulation capacity and physiological recovery following stress. Experimental evidence further indicates that short-term fasting-induced changes in heart-rate variability (HRV) are associated with interoceptive accuracy, supporting a functional link between autonomic regulation and interoceptive processing [22].

Overall, current evidence suggests that interoceptive processes may function as a modulatory interface between metabolic adaptations and emotional responses to IF. The psychological effects of fasting appear to depend not only on the underlying metabolic changes, but also on how such changes are perceived, interpreted, and regulated at the interoceptive level.

Further research integrating metabolic, autonomic, and interoceptive measures is needed to clarify the mechanisms underlying these associations and to better characterize individual variability in response to fasting.

### 3.4. Emotional Regulation and Fasting: Differential Outcomes

Current evidence indicates that the effects of intermittent fasting (IF) on emotional regulation are heterogeneous and influenced by the interaction between physiological adaptations and individual psychological characteristics. While IF has been consistently associated with metabolic benefits, its impact on affective processes appears to vary across individuals and contexts [25,26].

Recent literature suggests that emotional outcomes associated with fasting can be conceptualized across multiple domains, including mood regulation, stress responsivity, cognitive functioning, and vulnerability to psychopathology, supporting a multidimensional framework rather than a uniformly beneficial interpretation [3].

Some studies report that IF may be associated with improvements in perceived emotional regulation and reduced affective reactivity. These effects have been linked to increased tolerance of internal bodily signals and enhanced self-regulatory capacity, particularly in structured or supervised fasting conditions [27]. In such contexts, fasting may promote greater awareness of physiological states and facilitate adaptive emotional processing.

In contrast, other evidence indicates that IF may be associated with increased irritability, emotional lability, and cognitive rigidity, especially during early phases of adaptation or under conditions of prolonged or restrictive fasting [28,29]. These responses may reflect transient neuroendocrine changes, as well as individual differences in stress reactivity and interpretation of internal bodily signals.

Individual psychological traits appear to play a key moderating role. Higher levels of anxiety sensitivity, perfectionism, and need for control have been associated with less favorable emotional responses to fasting, whereas greater interoceptive confidence and flexible emotion regulation strategies may be associated with more adaptive outcomes [10,30]. Notably, perfectionistic traits may contribute to rigid behavioral patterns and, in some cases, to increased vulnerability to maladaptive fasting-related cognitions [30].

Clinical and developmental factors further contribute to variability in emotional responses. Individuals with a history of trauma, affective dysregulation, or disordered eating may exhibit increased vulnerability to negative psychological outcomes during fasting [31,32]. This is consistent with evidence indicating that fasting behaviors may overlap with or exacerbate maladaptive eating patterns in at-risk populations.

In addition, physiological adaptations to fasting, including changes in autonomic regulation, may influence emotional experience. For example, improvements in autonomic flexibility, reflected in changes in heart-rate variability, have been associated with enhanced emotional regulation capacity, although such effects appear to depend on individual and contextual factors [22].

ADF and prolonged fasting, which impose greater caloric restriction and more pronounced neuroendocrine perturbations, may carry a higher risk of negative affective outcomes, particularly in psychologically vulnerable individuals. TRE protocols, especially when aligned with the early circadian phase, appear associated with more stable emotional profiles in metabolically healthy populations. The 5:2 protocol occupies an intermediate position. These paradigm-specific considerations underscore the need for protocol-stratified analyses in future research.

Overall, emotional responses to IF should be interpreted as context-dependent and shaped by the interaction between metabolic adaptations, interoceptive processing, and psychological characteristics. This perspective highlights the limitations of generalized assumptions regarding the psychological effects of fasting and underscores the importance of considering individual variability.

A schematic overview of psychological profiles associated with differential emotional responses to IF is presented in Table 1. This framework is intended as a descriptive synthesis of patterns reported in the literature and should not be interpreted as a clinical classification.

Overall, available evidence indicates that emotional responses to IF are not uniform and are shaped by the interaction between metabolic changes and individual psychological characteristics. Further research integrating metabolic, psychological, and behavioral measures is needed to better characterize these differential outcomes.

Note: This framework is conceptual and descriptive in nature and should not be interpreted as a set of clinical recommendations.

### 3.5. Individual Moderating Factors

A growing body of evidence suggests that individual characteristics may substantially modulate the physiological and psychological responses to IF. Among these, biological sex, circadian timing, habitual physical activity, baseline metabolic status, and gut microbiome composition appear to be particularly relevant factors that have been insufficiently addressed in the existing literature.

Sex-related differences in the hormonal and metabolic responses to fasting have been increasingly documented. Emerging evidence suggests that women may exhibit a more pronounced HPA axis response to caloric restriction and may be more susceptible to fasting-induced alterations in reproductive hormones, eating-related cognitions, and mood [33]. Some clinical studies report that women following time-restricted eating protocols may experience cycle irregularities or increased affective lability, particularly under more restrictive conditions. These findings highlight the importance of sex-disaggregated analyses in future research and caution against generalizing fasting outcomes across sexes without appropriate stratification.

Circadian timing represents another key moderating variable. IF protocols that align caloric intake with the early active phase of the circadian cycle—a strategy commonly referred to as early time-restricted eating—have been associated with superior metabolic outcomes compared to protocols extending the eating window into the evening [14]. These circadian effects are mediated in part by clock-gene expression in metabolically active tissues and may interact with neuroendocrine systems involved in mood and arousal regulation, including cortisol rhythmicity and melatonin secretion [16]. The misalignment of fasting protocols with individual chronotype may therefore represent a source of variability in both metabolic and psychological outcomes.

Habitual physical activity and baseline metabolic status may further modulate the effects of IF. Individuals with higher cardiorespiratory fitness tend to exhibit greater metabolic flexibility, facilitating the shift to lipid-based energy utilization and potentially reducing adverse psychological responses during early fasting phases. Conversely, individuals with metabolic dysfunction, insulin resistance, or obesity may show attenuated ketogenic responses and differential endocrine adaptations, which may in turn influence interoceptive and emotional outcomes [12,13].

Finally, the potential modulatory role of the gut microbiome deserves attention as an emerging area of research. Preclinical and observational evidence suggests that IF may influence gut microbial composition and diversity, which in turn may affect brain function via the gut–brain axis, including through vagal afferent signaling, immune-mediated pathways, and microbial metabolite production such as short-chain fatty acids [19,21]. This bidirectional relationship may represent an additional layer of individual variability in psychological responses to fasting, although direct evidence from controlled human trials remains limited and causal inference is premature at this stage.

Taken together, these moderating factors underscore the complexity of individual responses to IF and highlight the need for stratified analyses accounting for sex, chronotype, physical activity level, and baseline metabolic status in future research. The integration of these variables into psychobiological models of IF may substantially improve the predictive validity of outcome frameworks and the clinical applicability of fasting recommendations.

### 3.6. Clinical Implications and Psychological Risks

Available evidence indicates that intermittent fasting (IF) is generally well tolerated in metabolically healthy individuals; however, its effects in clinically or psychologically vulnerable populations appear to be more variable. Some studies suggest that specific groups may be more susceptible to adverse psychological responses during fasting, including emotional dysregulation, increased somatic distress, and behavioral rigidity, particularly in the absence of adequate contextual support [28,31]. Importantly, recent clinical evidence indicates that structured fasting interventions may be associated with improvements in perceived stress and well-being in selected populations, whereas unsupervised or extreme fasting practices may lead to less predictable psychological outcomes [11,22].

Individuals with current or past eating disorders represent a population of particular interest in this context. Evidence suggests that fasting-related practices may overlap with or reinforce maladaptive behavioral patterns, including restrictive tendencies, cognitive rigidity, and heightened control over food-related behaviors [31,34]. These findings are consistent with observational data indicating that fasting behaviors may be associated with increased risk of eating disorder symptomatology in vulnerable populations [26].

Similarly, individuals with anxiety-related conditions may exhibit increased sensitivity to fasting-induced physiological changes. Alterations in hunger signals, autonomic activation, and energy availability may be experienced as distressing, potentially contributing to increased vigilance toward bodily sensations and heightened emotional reactivity [21,22]. In such cases, the subjective interpretation of physiological signals may play a relevant role in shaping the overall experience of fasting.

Individuals with a history of trauma may also present increased variability in response to fasting. Some evidence suggests that changes in bodily states associated with food restriction may interact with prior experiences related to control, safety, or bodily awareness, potentially influencing emotional regulation and stress responses [32,35].

In addition, emerging evidence suggests that fasting may not be psychologically neutral in affective disorders. Clinical and translational evidence suggests that fasting-related metabolic changes may influence mood-related pathways, although their clinical translation remains uncertain and context-dependent [26]. Relatedly, fasting may influence systemic immune function through immunometabolic mechanisms, including modulation of pro-inflammatory cytokine profiles and alterations in immune cell metabolism [36]. In individuals with low-grade chronic inflammation—a condition associated with depressive symptoms and reduced emotional resilience—fasting-induced immunomodulation could theoretically interact with mood-related pathways. However, direct evidence linking these immunometabolic effects to clinically relevant psychological outcomes remains preliminary, and further controlled studies are needed to evaluate this relationship.

Overall, the potential risks associated with IF appear to arise from the interaction between metabolic changes and individual differences in psychological functioning, including stress reactivity, interoceptive processing, and prior clinical history. These findings support a view of IF as a context-dependent intervention rather than a uniformly applicable approach.

From a clinical perspective, these observations suggest that the inclusion of psychological and behavioral factors may contribute to a more comprehensive understanding of individual responses to fasting. Variables such as eating-related attitudes, anxiety sensitivity, and emotion regulation strategies may be particularly relevant when evaluating suitability and tolerability.

In addition, interdisciplinary approaches involving collaboration between healthcare professionals from different fields have been proposed as a strategy to monitor both physiological and psychological responses during fasting interventions, particularly during early adaptation phases.

In summary, current evidence does not indicate that IF is inherently contraindicated in clinical populations; however, its effects appear to depend on individual characteristics and contextual factors. Further research is needed to better characterize risk profiles and to clarify the conditions under which fasting may be associated with either adaptive or maladaptive outcomes.

## 4. Discussion

This narrative review examined intermittent fasting (IF) as a psychobiological phenomenon, extending beyond its established role as a nutritional intervention. By integrating findings from metabolic research, neuroendocrinology, and psychological science, the present synthesis suggests that the effects of IF on emotional regulation may arise from the interaction between metabolic adaptations, endocrine signaling, and interoceptive processes.

Several considerations emerge from the available evidence. First, IF is consistently associated with metabolic and neuroendocrine adaptations, including changes in cortisol, insulin, and ketone body dynamics, which are involved in energy regulation and stress responsivity [4,18]. These physiological changes are closely linked to shifts toward lipid-based metabolism and may influence central nervous system functioning through pathways related to inflammation, mitochondrial activity, and synaptic plasticity. In addition, emerging evidence suggests that fasting-induced metabolic switching may also be associated with measurable changes in autonomic regulation and stress-related systems, including increases in heart-rate variability and reductions in perceived stress under controlled conditions [22]. At the same time, these adaptations are accompanied by alterations in internal bodily states that are processed through interoceptive networks, including the insular and cingulate cortices [7,8].

Second, the psychological effects associated with IF appear to vary substantially across individuals. Evidence indicates that factors such as interoceptive sensitivity, emotion regulation strategies, and trait-level characteristics may influence how fasting-related physiological changes are experienced. While some individuals report improved regulation of internal states, others may experience increased irritability, anxiety, or behavioral rigidity, particularly in the presence of pre-existing vulnerabilities [10,26]. These findings are consistent with recent integrative models describing fasting as a bidirectional intervention, potentially associated with both adaptive and maladaptive psychological outcomes depending on individual and contextual factors [3]. Collectively, these observations suggest that the psychological impact of IF cannot be inferred from metabolic outcomes alone.

The current literature presents several limitations that should be considered. A substantial proportion of studies focuses primarily on metabolic and physiological outcomes, with limited inclusion of validated psychological or interoceptive measures [11]. In addition, many studies are based on short-term interventions, which may not capture delayed or cumulative effects on emotional functioning [37]. This temporal limitation is particularly relevant, as initial responses to fasting may differ from longer-term adaptations.

Another limitation concerns the limited integration of biological and psychological domains within existing research designs. Studies often assess metabolic markers without parallel evaluation of psychological variables, or vice versa, making it difficult to clarify the pathways linking endocrine responses, interoceptive processing, and emotional outcomes. Furthermore, heterogeneity in fasting protocols and outcome measures reduces comparability across studies and may contribute to inconsistent findings.

Future research would benefit from integrative approaches combining metabolic, endocrine, and psychological measures within the same study designs. The inclusion of biomarkers such as cortisol, ketone bodies, inflammatory markers, and autonomic indices alongside validated assessments of interoception and emotion regulation may help clarify the relationships between physiological and subjective processes. Longitudinal studies are also needed to examine temporal dynamics and to distinguish between short-term adaptations and longer-term effects. In addition, future studies should systematically differentiate across IF paradigms—including TRE, ADF, the 5:2 protocol, and prolonged fasting—as the magnitude and character of metabolic and psychological effects are likely to vary across these protocols. Sex-stratified analyses represent a particularly important priority, given the evidence suggesting differential hormonal and psychological responses between men and women. Future research should also consider incorporating neuroimaging or psychophysiological methods to visually map the central correlates of fasting-related interoceptive and emotional processes, as this would substantially advance mechanistic understanding beyond what is currently possible through self-report and peripheral biomarkers alone.

From a broader perspective, the findings of this review support the interpretation of IF as a context-dependent intervention, whose psychological effects appear to depend on the interaction between physiological changes and individual characteristics. This perspective highlights the importance of considering both biological and psychological factors when evaluating the potential benefits and limitations of fasting, particularly in applied or clinical contexts.

**Figure 1:**
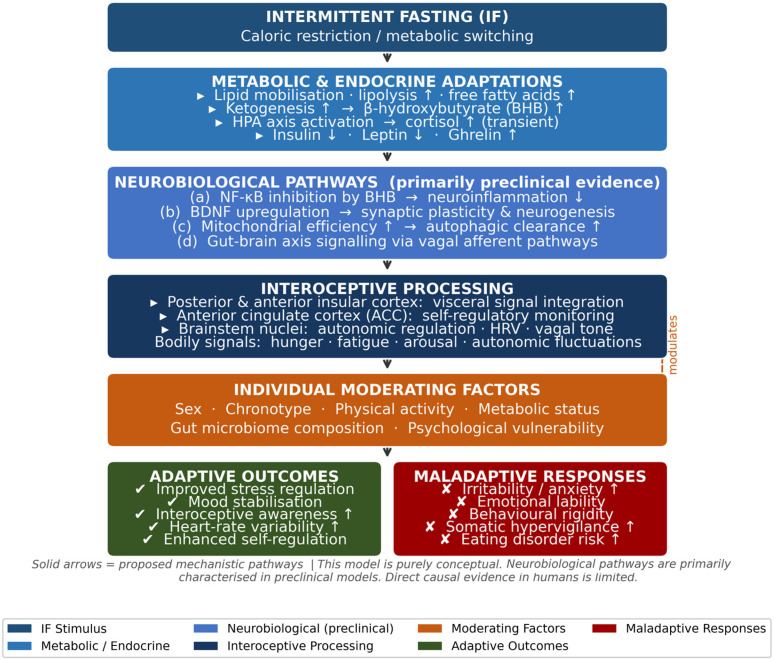
Conceptual framework of intermittent fasting as a psychobiological process linking metabolic adaptations to emotional outcomes. Intermittent fasting (IF) is represented as an initial metabolic and endocrine stimulus associated with lipid mobilization, ketone production (particularly β-hydroxybutyrate), HPA axis activation, and changes in insulin, leptin, and ghrelin signaling. These metabolic and neuroendocrine changes may influence central nervous system functioning through several proposed mechanisms, including: (a) neuroinflammatory modulation (via NF-κB pathway inhibition by β-hydroxybutyrate); (b) BDNF upregulation and synaptic plasticity; (c) improved mitochondrial efficiency and autophagic clearance; and (d) potential gut–brain axis signaling via vagal afferent pathways. Together, these processes contribute to variations in bodily signals—including hunger, fatigue, and autonomic fluctuations—which are processed through interoceptive mechanisms involving the insular cortex, anterior cingulate cortex, and brainstem nuclei. Interoceptive processing may influence subsequent emotional and behavioral responses, which vary substantially across individuals. Depending on individual differences in interoceptive sensitivity, stress reactivity, sex, chronotype, physical activity, and psychological vulnerability, these processes may be associated with either adaptive outcomes (e.g., improved regulation of internal states, increased HRV) or maladaptive responses (e.g., increased emotional instability or behavioral rigidity). Moderating individual factors are depicted as influencing the interoceptive-to-emotional processing pathway. This model is purely conceptual, based on the integration of findings discussed in the present review, and should not be interpreted as reflecting established causal pathways.

As illustrated in Figure 1, the effects of intermittent fasting may be conceptualized within a multilevel framework linking metabolic adaptations to psychological outcomes. In this model, fasting-induced metabolic and endocrine changes contribute to alterations in bodily signals, including hunger, fatigue, and autonomic fluctuations. These signals are subsequently processed through interoceptive mechanisms, which may influence emotional and behavioral responses. Depending on individual differences in interoceptive processing, stress reactivity, and psychological vulnerability, these processes may be associated with either adaptive outcomes, such as improved regulation of internal states, or less favorable responses, including increased emotional instability or behavioral rigidity. This framework is consistent with emerging evidence highlighting the role of interoception as a key mediator between physiological states and affective experience.

### Limitations

This review has several limitations that should be acknowledged. First, its narrative design does not allow for a systematic or quantitative evaluation of the available evidence. Although a structured search strategy was applied, the selection and interpretation of studies may be influenced by the authors’ perspective.

Second, the existing literature on intermittent fasting (IF) is characterized by substantial heterogeneity in study design, fasting protocols, populations, and outcome measures. This variability limits the comparability of findings and reduces the ability to draw definitive conclusions regarding the psychobiological effects of IF.

Third, a large proportion of studies in this field focuses primarily on metabolic and physiological outcomes, with limited inclusion of validated psychological or interoceptive measures. As a result, the integration of biological and psychological domains remains incomplete, and the mechanisms linking metabolic adaptations to emotional regulation are not yet fully established.

In addition, many available studies are based on short-term interventions, which may not capture longer-term psychological adaptations or delayed effects. The temporal dynamics of emotional responses to fasting therefore remain insufficiently understood.

Finally, individual moderating factors—including biological sex, chronotype, physical activity level, baseline metabolic status, and gut microbiome composition—are often not systematically accounted for in existing research, along with psychological traits, clinical history, and contextual factors. The lack of sex-disaggregated analyses in particular represents a significant gap, as available evidence suggests that women may respond differently to IF at both the hormonal and psychological level. Similarly, the absence of consistent differentiation across fasting paradigms (e.g., TRE vs. ADF vs. prolonged fasting) limits the specificity of conclusions and the generalizability of findings across populations.

Taken together, these limitations highlight the need for more integrative, longitudinal, and methodologically consistent research to better characterize the psychobiological effects of intermittent fasting.

## 5. Conclusions

Intermittent fasting (IF) should be conceptualized not only as a metabolic intervention, but as a multidimensional psychobiological process involving the interaction between metabolic adaptations, neuroendocrine signaling, and interoceptive mechanisms. The present review highlights that fasting-related physiological changes extend beyond lipid metabolism and insulin regulation, encompassing processes that are closely linked to emotional regulation and subjective experience.

Current evidence suggests that IF may act as a controlled metabolic stressor capable of modulating both physiological and psychological systems. However, its effects on emotional regulation are not uniform and appear to depend on individual differences in stress responsivity, interoceptive processing, and psychological vulnerability. This variability underscores the limitations of generalized assumptions regarding the psychological benefits of fasting.

The findings of this review support a context-dependent interpretation of IF, in which metabolic and psychological outcomes emerge from the dynamic interaction between biological adaptations and individual characteristics. In this framework, interoception may represent a key mediating process linking physiological changes to emotional and behavioral responses.

Despite increasing interest in this field, current literature remains limited by a lack of integrative study designs combining metabolic, endocrine, and psychological measures. Future research should prioritize longitudinal and multidisciplinary approaches to better characterize temporal dynamics, identify differential response profiles, and clarify the mechanisms underlying individual variability.

Overall, a more integrated perspective is needed to fully understand the psychobiological effects of intermittent fasting and to inform its application in both clinical and non-clinical contexts.

## Figures and Tables

**Table 1:** Conceptual psychological profiles associated with differential emotional responses to intermittent fasting. This table is purely descriptive and is based on qualitative patterns emerging from the reviewed literature. It does not constitute a validated clinical instrument and should not be used for individual risk stratification or clinical decision-making. Categories are not mutually exclusive and represent simplified heuristic constructs for illustrative purposes only.

Psychological Profile	Relative Risk Level	Interpretative Consideration
High Interoceptive Awareness	Low	May be associated with more adaptive emotional regulation
Rigid Perfectionism	Moderate to High	May be associated with increased behavioral rigidity
Anxiety Sensitivity/Trauma history	High	Associated with increased vulnerability to emotional dysregulation
Current or Past Eating Disorder	Very high	Generally associated with poorer tolerance to fasting

## Data Availability

No new data were created or analyzed in this study. Data sharing is not applicable to this article.

## Abbreviations

The following abbreviations are used in this manuscript:

IF	Intermittent Fasting
HPA	Hypothalamic–Pituitary–Adrenal
ANS	Autonomic Nervous System
HRV	Heart-Rate Variability
TRE	Time-Restricted Eating
BHB	β-hydroxybutyrate
